# Diagnosis of coronary epicardial and microvascular spasm using pressure wire before and after spasm provocation

**DOI:** 10.1093/ehjcr/ytaf292

**Published:** 2025-06-23

**Authors:** Hiroyuki Omori, Makoto Iwama, Takahiko Suzuki, Toshiyuki Noda

**Affiliations:** Department of Cardiology, Gifu Prefectural General Medical Center, Japan 4-6-1 noishiki, Gifu 500-8717, Japan; Department of Cardiovascular Medicine, Toyohashi Heart Center, Aichi 441-8530, Japan; Department of Cardiology, Gifu Prefectural General Medical Center, Japan 4-6-1 noishiki, Gifu 500-8717, Japan; Department of Cardiovascular Medicine, Toyohashi Heart Center, Aichi 441-8530, Japan; Department of Cardiology, Gifu Prefectural General Medical Center, Japan 4-6-1 noishiki, Gifu 500-8717, Japan

An 80-year-old male presented with new-onset early-morning chest pain at rest. Electrocardiography showed no ST-segment changes, and troponin I was negative. He had a history of percutaneous coronary intervention for a severe lesion in the ostial left anterior descending artery in 2011.

Coronary angiography (CAG) revealed intermediate stenosis in the mid-right coronary artery (see Supplementary material online, *Figure S1A*, *Videos S1* and *S2*). Coronary physiology was assessed using a pressure-temperature sensor guidewire (PressureWire X; Abbott Vascular). Hyperaemia was induced by intracoronary bolus injection of 2 mg nicorandil.^1^ Initial measurements indicated normal values with a fractional flow reserve (FFR) of 0.92, coronary flow reserve (CFR) of 2.4, and index of microcirculatory resistance (IMR) of 24 (*Figure 1B*).

**Figure 1:**
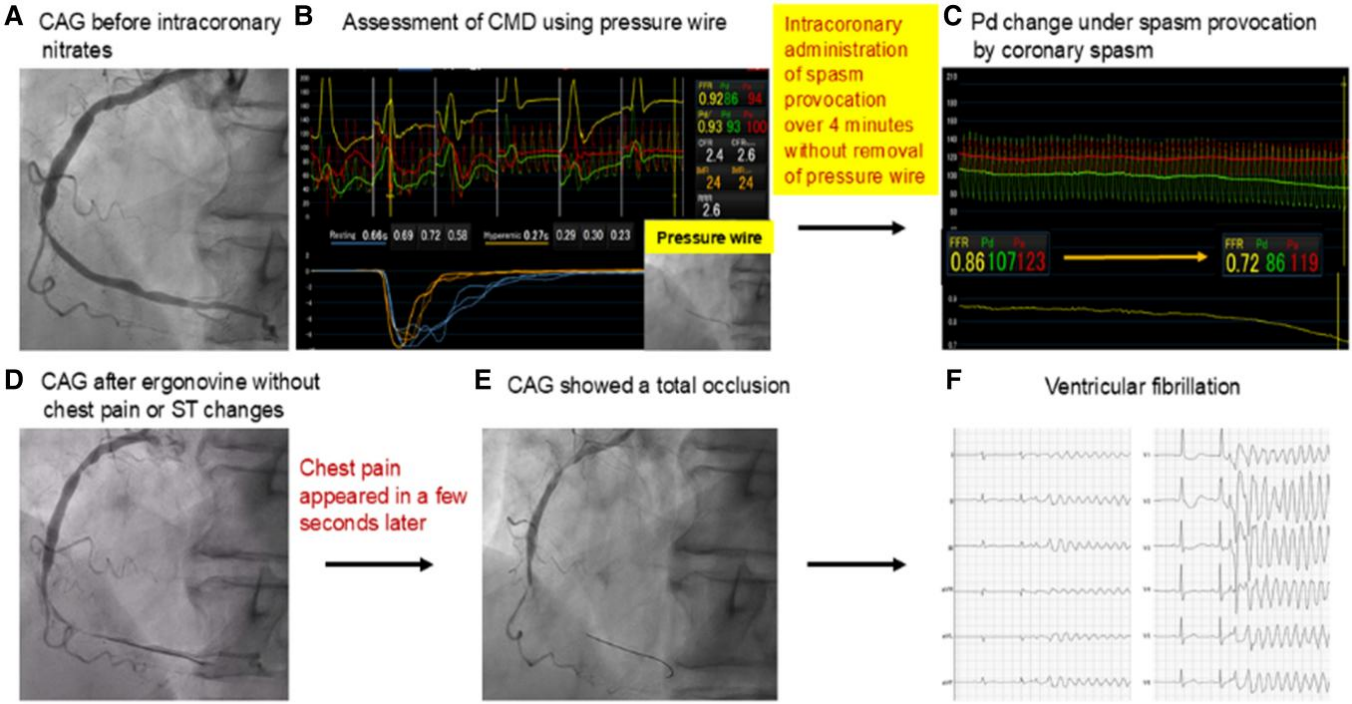
Assessment of ischemia with non-obstructive coronary artery disease. (*A*) Coronary angiography showed intermediate stenosis in the mid-right coronary artery before the administration of intracoronary nitroglycerin. (*B*) Initial coronary microvascular dysfunction assessment showed normal values. (*C*) Pressure wire measurements demonstrated a decrease in Pd from 107 mmHg to 86 mmHg under spasm provocation. (*D*) Coronary angiography revealed 99% coronary luminal narrowing without chest pain or ST-segment changes. (*E*) A few seconds later, chest pain appeared, and coronary angiography revealed total occlusion. (*F*) The patient developed ventricular fibrillation.

For spasm provocation, intracoronary ergonovine was administered at 5 μg/min for 4 min (total 20μg).^1^ To detect spasms early, the pressure wire remained in place to monitor distal coronary artery pressure (Pd) changes. After 3 min of ergonovine administration, Pd gradually decreased from 107 to 86 mmHg (*Figure 1C*). Although the patient reported no chest pain or ST-segment changes, CAG was performed due to suspected spasm, revealing 99% coronary luminal narrowing (*Figure 1D*, Supplementary material online, *Video S3*). A few seconds later, the patient developed chest pain, and following CAG showed total occlusion (*Figure 1E*, Supplementary material online, *Video S4*). Before nitrates were being administered, the patient developed ventricular fibrillation (*Figure 1F*). Immediate electrical defibrillation restored sinus rhythm. Intracoronary nitrates (300μg) were administered, and subsequent CAG showed resolution of the coronary spasm, although with slow flow (*Figure 2A*, Supplementary material online, *Video S5*). The patient’s chest pain persisted without ST-segment changes (*Figure 2B*). Suspecting microvascular spasm, we reassessed coronary microvascular dysfunction (CMD) using pressure wire measurements for increased diagnostic accuracy. Results showed an FFR of 0.94, CFR of 7.1, and IMR of 34, indicating an increased IMR (*Figure 2C*). Notably, the resting mean transit time [Tmn] prolonged from 0.66 s to 2.65 s, while the hyperaemic Tmn increased from 0.27 s to 0.37 s (see Supplementary material online, *Table S1*). The patient’s chest pain resolved after intracoronary administration of nicorandil, which improved the slow flow (see Supplementary material online, *Video S6*). He remained asymptomatic while taking oral nicorandil.

**Figure 2:**
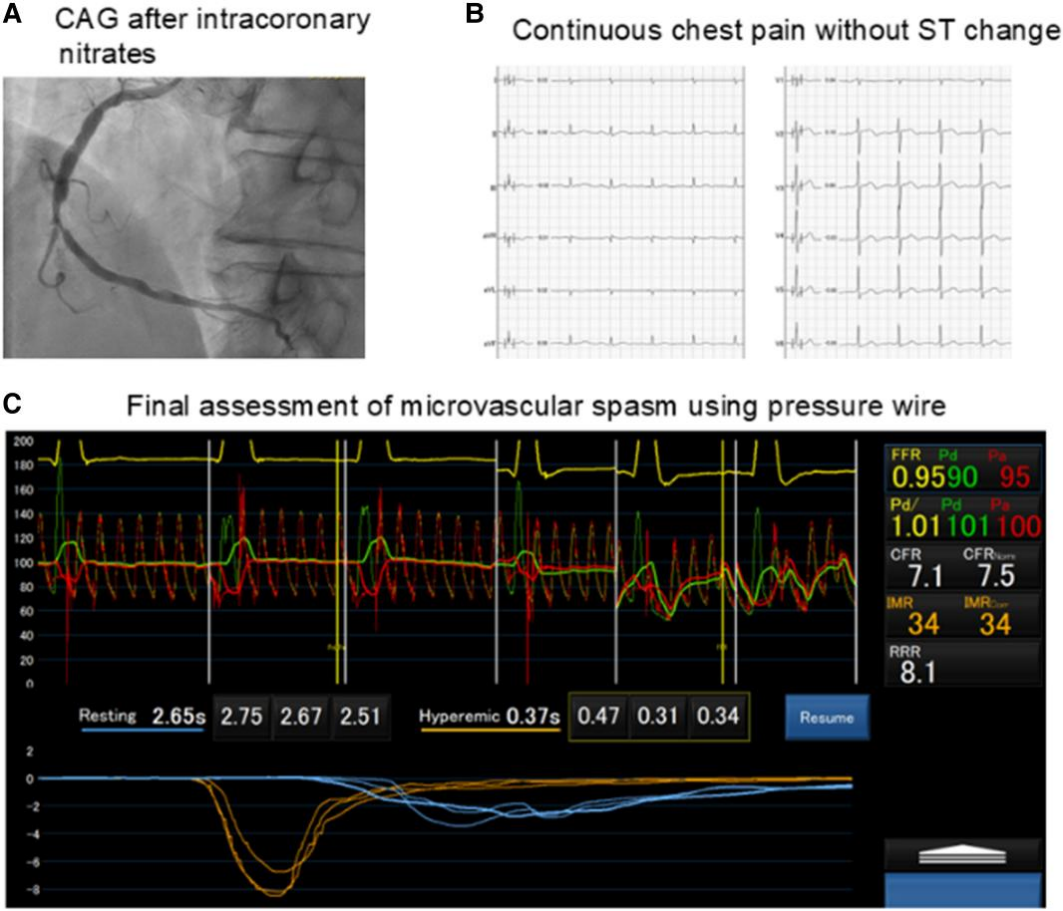
Assessment of microvascular spasm using pressure wire. (*A*) Coronary angiography after intracoronary nitrate administration showed resolution of the coronary spasm, though with slow flow. (*B*) Electrocardiography showed no ST-segment changes with chest pain. (*C*) Final assessment of coronary microvascular dysfunction numerically showed microvascular spasm.

In this patient, an initial IMR of 24 ruled out structural CMD. Severe epicardial spasm was observed at mild-to-moderate plaque burden, likely precipitated by endothelial dysfunction. Notably, real-time monitoring of Pd alerted us to impending severe spasm before chest pain or ST changes emerged. Even after epicardial spasm was relieved, the patient continued to experience chest pain. Prolonged Tmn likely resulted from microvascular spasm. Appropriate vasodilator therapy with nicorandil alleviated the patient’s symptoms and prevented recurrence.

Although 2024 ESC guidelines recommend the use of acetylcholine, ergonovine remains an alternative.^2^ Nicorandil was chosen as the hyperaemic agent because it has fewer adverse effects than adenosine.^1,3^ Its short half-life may also help minimize interference with subsequent spasm testing.

This case underscores the diagnostic value of simultaneous epicardial and microvascular functional assessments. Keeping the pressure wire in place during provocation enabled early recognition of epicardial spasm (indicated by a drop in Pd) and facilitated prompt treatment. Additionally, it identified coexisting microvascular spasm, highlighting the need for tailored therapy.

## Lead author biography


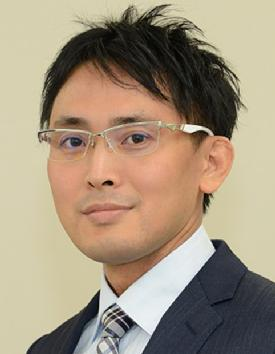


I am an interventional cardiologist from Japan, with a primary research focus on coronary physiology, coronary imaging, and endovascular therapy.

## Supplementary Material

ytaf292_Supplementary_Data

## Data Availability

All data relevant to this case report are included in the article. Additional data or materials can be made available by the corresponding author upon reasonable request, as per the journal’s data availability policy.

## Notes

**Consent:** Written informed consent was obtained from the patient prior to publication, in full accordance with the Committee on Publication Ethics guidelines.

**Funding:** None declared.

## References

[1] Hokimoto S, Kaikita K, Yasuda S, Tsujita K, Ishihara M, Matoba T, et al JCS/CVIT/JCC 2023 guideline focused update on diagnosis and treatment of vasospastic angina (coronary spastic angina) and coronary microvascular dysfunction. J Cardiol 2023;82:293–341. 37597878 10.1016/j.jjcc.2023.06.009

[2] Vrints C, Andreotti F, Koskinas K, Rossello X, Adamo M, Ainslie J, et al 2024 ESC Guidelines for the management of chronic coronary syndromes. Eur Heart J 2024;45:3415–3537. 39210710 10.1093/eurheartj/ehae177

[3] Seitz A, Feenstra R, Konst R, Pereyra V, Beck S, Beijk M, et al Acetylcholine rechallenge: a first step toward tailored treatment in patients with coronary artery spasm. JACC Cardiovasc Interv 2022;15:65–75. 34991826 10.1016/j.jcin.2021.10.003

