# Supplementary material for: Diagnosis of coronary epicardial and microvascular spasm using pressure wire before and after spasm provocation
Source: Eur Heart J Case Rep. 2025 Jun 23;9(7):ytaf292. doi: 10.1093/ehjcr/ytaf292 (PMC12246919; doi:10.1093/ehjcr/ytaf292)
Supplement: ytaf292_Supplementary_Data [file ytaf292_supplementary_data.zip › Supplenetal Table.docx]

**Supplementary table**

|  | Before provocation | After provocation |
| --- | --- | --- |
| Chest pain | - | + |
| ECG change | - | - |
| Resting Tmn (seconds) | 0.66 | 2.65 |
| Hyperemic Tmn (seconds) | 0.27 | 0.37 |
| IMR | 24 | 34 |
| CFR | 2.4 | 7.1 |
| FFR | 0.92 | 0.94 |
